# Cardiac power integral: a new method for monitoring cardiovascular performance

**DOI:** 10.1002/phy2.159

**Published:** 2013-11-19

**Authors:** Audun E Rimehaug, Oddveig Lyng, Dag O Nordhaug, Lasse Løvstakken, Petter Aadahl, Idar Kirkeby-Garstad

**Affiliations:** 1 Department of Circulation and Medical Imaging, Norwegian University of Science and TechnologyTrondheim, Norway; 2 Clinic of Anesthesiology and Intensive Care, Trondheim University HospitalTrondheim, Norway; 3 Unit of Comparative Medicine, Norwegian University of Science and TechnologyTrondheim, Norway; 4 Department of Thoracic Surgery, Trondheim University HospitalTrondheim, Norway; 5 Department of Thoracic Anesthesiology and Intensive Care, Trondheim University HospitalTrondheim, Norway

**Keywords:** Cardiac power output, left ventricular energy production, stroke work

## Abstract

Cardiac power (PWR) is the continuous product of flow and pressure in the proximal aorta. Our aim was to validate the PWR integral as a marker of left ventricular energy transfer to the aorta, by comparing it to stroke work (SW) under multiple different loading and contractility conditions in subjects without obstructions in the left ventricular outflow tract. Six pigs were under general anesthesia equipped with transit time flow probes on their proximal aortas and Millar micromanometer catheters in their descending aortas to measure PWR, and Leycom conductance catheters in their left ventricles to measure SW. The PWR integral was calculated as the time integral of PWR per cardiac cycle. SW was calculated as the area encompassed by the pressure–volume loop (PV loop). The relationship between the PWR integral and SW was tested during extensive mechanical and pharmacological interventions that affected the loading conditions and myocardial contractility. The PWR integral displayed a strong correlation with SW in all pigs (*R*^2^ > 0.95, *P <* 0.05) under all conditions, using a linear model. Regression analysis and Bland Altman plots also demonstrated a stable relationship. A mixed linear analysis indicated that the slope of the SW-to-PWR-integral relationship was similar among all six animals, whereas loading and contractility conditions tended to affect the slope. The PWR integral followed SW and appeared to be a promising parameter for monitoring the energy transferred from the left ventricle to the aorta. This conclusion motivates further studies to determine whether the PWR integral can be evaluated using less invasive methods, such as echocardiography combined with a radial artery catheter.

## Introduction

Recently, a measure of cardiac effect (energy/time), known as the cardiac power output (CPO), has been shown to strongly correlate with clinical outcomes after acute cardiac shock (Fincke et al. 2004), chronic heart failure (Cohen-Solal et al. 2002), and a broad spectrum of acute cardiac diseases (Williams et al. 2001; Fincke et al. 2004; Mendoza et al. 2007). CPO corresponded better to the patient outcome than blood pressure or blood flow, indicating that the hydraulic power transferred from the heart to the vasculature may be a more fundamental hemodynamic parameter than pressure or flow alone (Fincke et al. 2004). CPO is, however, by most existing technologies in use, only give a measurement once a minute, not be able to adjust for respiratory variations, and require relatively invasive procedures. The existing hemodynamic parameters in clinical practice today are summarized in Table 1.

**Table 1:** Hemodynamic parameters available today

	Method	Advantages	Disadvantages
Mean arterial pressure (MAP)	Through intraarterial catheter linked to manometer	Easily available	Influenced by many noncardiac factors Contains hardly any information about flow and oxygen transportation
Cardiac output (CO)	Pulmonary artery catheter (PAC), pulse contour analysis	Closely related to oxygen transportation	Demands invasive procedures and/or equipment often not easily available
Cardiac power output (CPO)	The product of MAP and CO	Contains information about both pressure and flow, the total energy transfer from the heart	Demands all the equipment to measure both MAP and CO When using PAC, only available once a minute and difficult to correct for respiratory cycle

We find these parameters insufficiently informative and available.

Cardiac power (PWR) is the product of blood pressure and flow in the proximal aorta. A continuous PWR curve may be constructed by multiplying instantaneously measured aortic flow and pressure curves (Kass and Beyar 1991); clinically, the combination of the flow measured by ultrasound and invasively measured blood pressure has been used (Sharir et al. 1994; Nakayama et al. 1998; Schmidt et al. 1999; Segers et al. 2002). CPO, calculated as the product of cardiac output (CO) and mean arterial pressure (MAP), is a representation of the *mean* hydraulic power. The time integral under the PWR curve (PWR integral) represents the *total* hydraulic power (= mean hydraulic power + oscillatory power) transferred from the heart to the proximal aorta. With the oscillatory power accounting for approximately 15% of the total power (Westerhof et al. 2005), we suggest that the PWR integral may be a more direct, more easily accessed, and more precise measurement of the hydraulic power transferred from the heart to the vasculature than CPO.

PWR is relatively independent of afterload, but strongly dependent on preload (Kass and Beyar 1991). The aim of this study was to further validate the PWR integral as a marker of left ventricular energy transfer to the vasculature during alterations in loading conditions and contractility. The notion that energy produced in the heart is fully transferred to the aorta is described in textbooks (Westerhof et al. 2005), but how loading conditions and alterations of contractility affect this transfer has not been sufficiently investigated. We compared the PWR integral to stroke work (SW) calculated as the area encompassed by the pressure–volume (PV) loop obtained by a left ventricular conductance catheter, the gold standard for quantifying cardiac function (Kass et al. 1986; Burkhoff et al. 2005). CPO appears to correspond well to changes in the SW (Post et al. 2009). As the SW and PWR integral are expressions of total power, whereas the CPO is an expression of mean power (Westerhof et al. 2005), we find it plausible that the PWR integral will follow SW at least as well as CPO and on a stroke-to-stroke basis.

We used a highly invasive but reliable method to measure the PWR. Our hypothesis was that the PWR integral would follow SW across different loading and contractility conditions, and across individuals. If the PWR integral is validated, we would like to further develop the method so that PWR can be measured with minimally invasive methods such as transesophageal or transthoracic ultrasound.

## Material and Methods

Six male Noroc pigs (hybrid of ¼ Duroc, ¼ Yorkshire, and ½ Norwegian landrace) weighing 25–30 kg were used to test our hypothesis. The protocol was approved by the local steering committee of the Norwegian Experimental Animal Board. All the animals received humane care in compliance with the European Convention on Animal Care.

### Anesthesia and medical preparations

The animals were premedicated with intramuscular injections of azaperone 4 mg/kg and ketamine 20 mg/kg. Before the operations, the pigs were cleaned and weighed. Anesthesia was then induced through i.v. access on the external ear of the animals with fentanyl 0.04 mg/kg, ketamine 10 mg/kg, pentobarbital 10 mg/kg, and atropine 1 mg. Respiratory control was achieved with ventilation through a tracheostomy tube. The respirator was set in volume-controlled mode with FiO_2_ = 0.6. The tidal volume was adjusted to obtain normocapnia and a Po_2_ of ≥12 kPa. Anesthesia maintenance was achieved with fentanyl 0.02 mg kg^−1^ h^−1^ and midazolam 0.3 mg kg^−1^ h^−1^, and the infusion rate was eventually increased based on the clinical response. Intravascular volume was maintained by infusing acetated Ringer's solution and polyhydroxy methyl starch, and 50-mL boluses of Ringer's solution were added when indicated by central venous pressure (CVP), heart rate, and systemic blood pressure. A 150-mg bolus of amiodarone was administered intravenously (IV) to prevent arrhythmias. Hexamethonium 20 mg/kg was administered IV to avoid reflex changes in hemodynamics during interventions. Isoflurane gas anesthesia was administered as needed during shorter periods.

### Surgical preparation

A central venous line was inserted in the left jugular vein for infusions and in the right jugular vein for CVP measurements. Urine production was monitored through cystostomy and bladder catheterization. A catheter was inserted into the right brachial artery for continuous blood pressure monitoring and blood gas sampling. After a sternotomy, a combined pressure conductance catheter was inserted in the left ventricle from the right internal carotid artery, and a micromanometer catheter was inserted in the descending aorta via the left carotid artery. A transit time flow probe was mounted on the ascending aorta. A rubber band was placed around the inferior caval vein for preload reductions, and a balloon catheter was inserted in the ascending aorta via the right femoral artery for afterload augmentation. In addition, 5000 IU heparin was administered IV as a prophylaxis to thrombus formation.

### Measurements and Calculations

In-house software instantaneously recorded the following variables:

1. electrocardiogram (ECG), left ventricular pressure (LVP), left ventricular volume (LVV), and SW using a conductance catheter Leycom Sigma 5DF (CD Leycom, Zoetermeer, The Netherlands),
2. aortic blood pressure (ABP) using a Millar catheter connected to a CPU-2000 unit (Millar, Houston, TX), and
3. aortic flow and CO from a CardioMed CM4000 transit time flow probe (Medistim, Oslo, Norway).

Because both the transit time flow probe and Millar catheter have a sampling frequency of 1000 Hz, PWR could easily be calculated by the in-house software as the continuous product of flow and pressure. The time integral of the PWR for each cardiac cycle was calculated directly by the in-house software using the numeric integration IV block in Labview. The PWR integral was then compared with SW, which was measured as the area encompassed by the PV loop from the conductance catheter in the left ventricle. The volume measured by the conductance catheter was calibrated using alpha correction once per animal, in accordance with other studies (Szwarc et al. 1994). This alpha correction calibrates the stroke volume measurement from the conductance catheter using the measurement from the transit time probe.

We wanted to test the relationship between SW and the PWR integral both during mechanical alterations in loading conditions and during new steady-state conditions using pharmacological interventions. To reduce random variation and signal disturbances, the ventilator was disconnected during the measurements. For each measurement, the data sets from 10 cardiac cycles were collected. Between the measurements, the ventilator was reconnected, and the animal was allowed to stabilize.

**Figure 1:**
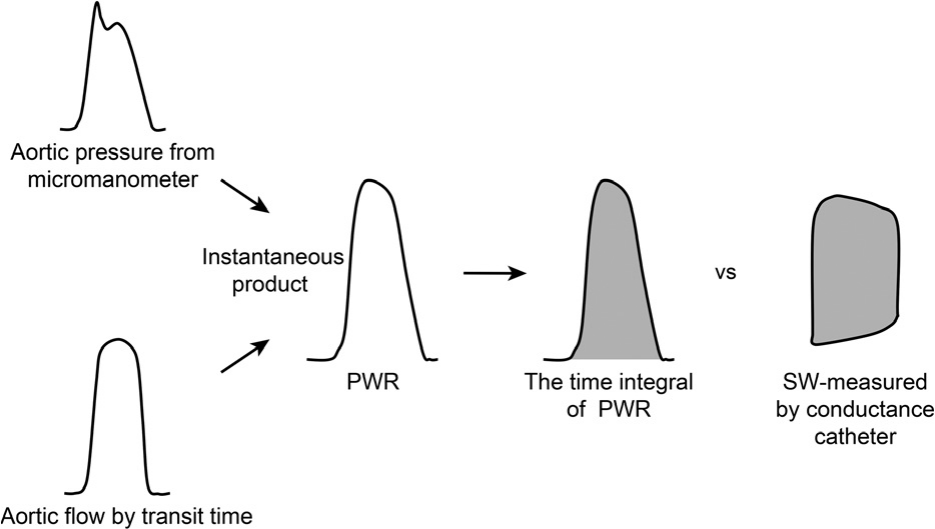
PWR was determined by multiplying the aortic pressure by aortic flow. Aortic pressure was measured with a micromanometer in the descending aorta. Aortic flow was measured with a transit time flow meter in the ascending aorta. The PWR integral was calculated as the time integral for each cardiac cycle. The PWR integral was then compared with SW, which was measured using a conductance catheter in the left ventricle.

**Figure 2:**
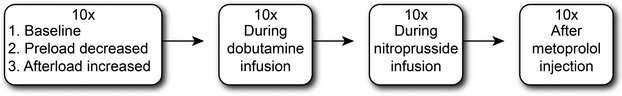
The measurement order. Each measurement contained 10 cardiac cycles. First, we recorded 10 sets of measurements, where each set consisted of one baseline measurement, one during reduced preload and one during increased afterload. Thereafter, 10 measurements were recorded during dobutamine infusion, 10 during nitroprusside infusion, and 10 after a metoprolol injection. The ventilator was disconnected during each measurement, and the animal was allowed to stabilize between every measurement.

A summary of the relationship between measured variables is illustrated in Figure 1, and the order of measurements is illustrated in Figure 2. The mechanical interventions were performed first to avoid the effects of residual pharmacological interventions. We gathered 10 sets consisting of one baseline measurement, one measurement during mechanically reduced preload, and one measurement during mechanically increased afterload, in that order. For mechanical preload reduction, we used a rubber band around the inferior vena cava, tightening the band enough to achieve at least a 20% reduction in CO at the start of the recorded set. For increased afterload, we used an embolectomy catheter with a 2-mL balloon that was placed in the distal descending aorta via the right femoral artery. The recordings during the reduced preload and during the increased afterload were performed immediately after the intervention to avoid the effects of compensation mechanisms.

Thereafter, we applied pharmacological interventions to achieve new steady-state conditions, using agents that affected loading conditions and/or contractility. We recorded 10 measurements consisting of 10 cardiac cycles in each condition before moving on to the next: first, during the infusion of dobutamine, 2.5 μg kg^−1^ min; second, during the infusion of sodium nitroprusside, 0.5 μg kg^−1^ min; and third, after a bolus injection of metoprolol, 0.5 mg/kg.

Under six different conditions (using 10 measurements from each and 10 cardiac cycles in each measurement), we gathered a total of 600 (10 × 10 × 6) pairs of synchronously measured SW and PWR integral values per animal.

At the end of the experiment, the animal was euthanized while still under general anesthesia, using 40 mL of pentobarbital 100 mg/mL.

### Analysis and statistics

The recorded files were refined using the previously mentioned in-house software before the results were exported to SPSS (IBM Corp. Released 2011. IBM SPSS Statistics for Windows, Version 20.0; IBM Corp., Armonk, NY) for plotting and analysis. Recordings with obvious technical malfunctions were excluded.

**Figure 3:**
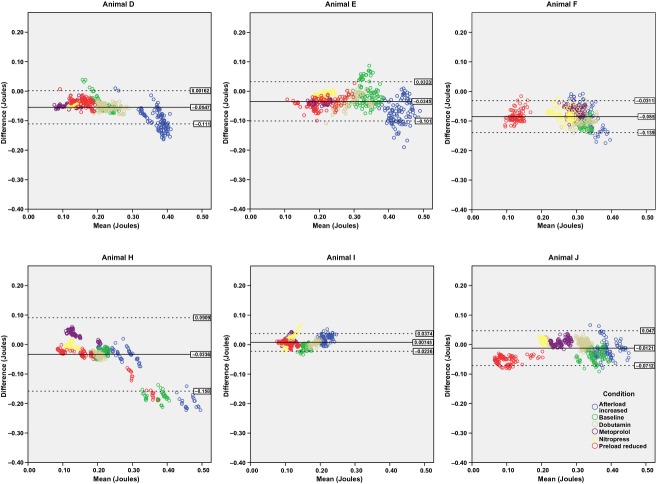
A Bland Altman plot for each animal individually. The PWR integral subtracted from SW is on the *y*-axis, the mean of SW and the PWR integral is on the *x*-axis. Each marker represents one cardiac cycle, the markers are color coded by the condition of the animal.

The relation between SW and the PWR integral was compared in a linear plot and evaluated using Pearson's correlation coefficient for each animal individually. We also added a linear approximation to the relation for all the material and for each individual animal using regression analysis, assuming no intercept, as SW = 0 would necessarily yield PWR = 0. Quadratic regression lines were tested, but did not yield a significantly better fit. The relation was also analyzed using a Bland Altman plot for each animal individually, see Figure 3. In the Bland Altman plots, the PWR integral was subtracted from the SW on the *y*-axis, and the mean of the PWR integral and the SW on the *x*-axis.

Finally, we investigated the fit of the data in a mixed linear model, considering the animal a random effect and the intervention a fixed effect. This process allowed us to investigate if and how the single animal or the interventions would affect the relation between SW and the PWR integral. In addition, in this study, no intercept was assumed for both the random and fixed effects.

## Results

**Figure 4:**
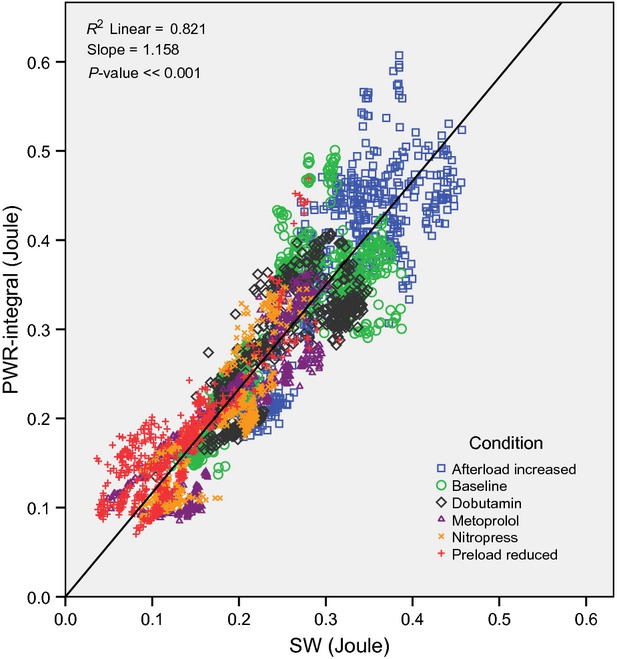
Correlation plot for all the animals in the same chart, comparing SW to the PWR integral, with the units in Joules on both axes. Each mark is one cardiac cycle, and each animal is coded with an individual symbol. All conditions are included in the plot: baseline, reduced preload, increased afterload, dobutamine infusion, nitroprusside infusion, and metoprolol bolus injection, each coded with a separate color. The linear regression line for the full material was added and calculated with the assumption that SW = 0 will yield PWR = 0.

All six animals were included in the analysis and 3450 paired measurements were obtained. All six animals displayed a close correlation between SW and the PWR integral with a Pearson correlation coefficient range 0.95–0.99 (*P* < 0.01) when calculated for each animal separately. The slope of SW versus the PWR integral relation varied between the animals (range 0.95–1.33). The correlation coefficients and the slope of the linear approximation with a 95% confidence interval are shown in Table 2. A linear plot of SW versus the PWR integral for the entire material is shown in Figure 4. The linear regression line for the entire material revealed a slope of 1.158. Quadratic regression lines were tested, but did not yield a significantly better fit. All conditions are included in the plot, each coded with a different color: baseline, reduced preload, increased afterload, dobutamine infusion, nitroprusside infusion, and metoprolol bolus injection. Based on the assumption that SW = 0 necessarily implies that PWR = 0, both the correlation and linear regression were calculated without a constant.

**Table 2:** SW to PWR integral correlation

Animal	Correlation *R*^2^ PWR integral–SW	Slope PWR–SW
D	0.993 (<0.01)	1.29 (1.281–1.300)
E	0.988 (<0.01)	1.12 (1.110–1.130)
F	0.987 (<0.01)	1.33 (1.320–1.345)
H	0.955 (<0.01)	1.24 (1.220–1.264)
I	0.991 (<0.01)	0.95 (0.939–0.954)
J	0.989 (<0.01)	1.03 (1.019–1.037)

The *P*-value is given in parentheses, and the slope of the linear regression line is given with a 95% confidence interval. Animals A–C were pilots, animal G was excluded from analysis due to technical failure.

The Bland Altman plot of each animal individually in Figure 3 illustrates a stable relation between the PWR integral and SW across all conditions, with the exception of animal H. This exception is discussed below. The mean of the difference SW minus PWR integral ranges from −0.085 in animal F to 0.007 in Animal I. The standard deviation of the same difference ranges from 0.015 in animal I to 0.062 in animal H.

The mixed linear model results are displayed in Table 3. We used the baseline condition as a reference and considered the animal a random effect, whereas we considered the interventions a fixed effect. The assumption of no intercept was also applied here. All interventions resulted in a significant change in the slope when compared to the baseline condition, with the exception of the condition with mechanically increased afterload (*P =* 0.092). The shallowest slope was found after metoprolol injection and during nitroprusside infusion. We also attempted a quadratic regression in this model, but it did not yield a significantly better fit. The correlation between SW and the PWR integral was strong in all conditions. The difference in slope between the animals as a random effect was not significant (*P =* 0.115).

**Table 3:** Mixed linear analysis

Condition	Slope	*P*-value
Baseline	1.19 (1.06–1.31)	
Afterload increased	1.18 (1.05–1.30)	0.092
Preload reduced	1.28 (1.16–1.41)	<0.001
Dobutamine	1.14 (1.01–1.26)	<0.001
Metoprolol	1.07 (0.94–1.20)	<0.001
Nitroprusside	1.10 (0.97–1.22)	<0.001

Baseline was used as the reference condition. The slope of the regression for each condition was significantly different from the slope of the reference condition, with the exception of the increased afterload condition. The lowest slope was associated with the metoprolol infusion and nitroprusside infusion conditions.

**Figure 5:**
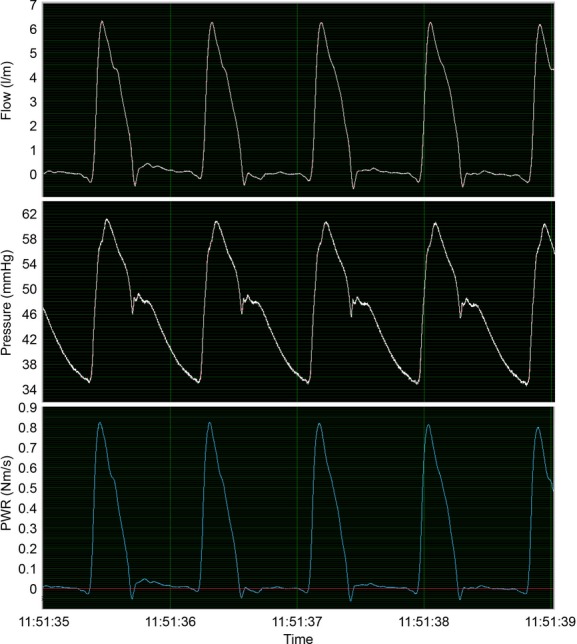
Pressure data from the aortic micromanometer is illustrated on top, displayed in mmHg; the flow from the transit time probe on the ascending aorta is displayed in the middle, and the resulting product PWR is displayed at the bottom. The software calculated the integral of the PWR curve per cycle. Because flow has a much higher relative variation during a cycle than pressure, flow will dominate the shape of the PWR curve. In addition, because flow is practically zero during diastole, the diastolic pressure will not have much effect on the PWR.

As shown in Figure 5, we observed that the PWR curve and the flow curve had similar shapes when compared with the pressure curve. This is because flow had a much higher relative variation during a cycle than pressure in our research animals and therefore dominated the shape of the PWR curve, which is the product of pressure and flow. The software calculated the integral of the PWR curve per cycle. Because the flow is practically zero during diastole, the diastolic pressure will not have much effect on the PWR integral.

## Discussion

Our primary aim was to validate the PWR integral as a marker of cardiac energy transfer to the vasculature under varying loading conditions and inotropic states. The PWR integral achieved by invasive measurements on the proximal aorta was compared stroke to stroke with the SW calculated from the LVP–volume loop during a series of mechanical and pharmacological interventions. All six animals presented high individual correlations between SW and the PWR integral. The scatter plot shows that we did not have any outliers that might represent a risk in the clinical use of the PWR integral. SW and the PWR integral followed one another closely during all interventions described and across the animals.

The Bland Altman plots in Figure 3 illustrate a stable relation between the SW and the PWR integral, with a slightly higher value of the PWR integral, consistent with the findings of the regression analysis. In Animal H the Bland Altman plot shows a higher difference at higher values. In this case we found the reason to be an induced mitral insufficiency. The cardiac cycles from early in the experiment are to the right in the diagram. After these we found a sudden drop in aortic flow, but an increase in stroke volume measured by the conductance catheter, a leftward leaning isovolumetric contraction curve in the PV loop, and a decreased end systolic volume. All these findings indicate that Animal H had a sudden mitral insufficiency causing this shift in the SW-to-PWR integral relation.

We had no specific expectations regarding how the different loading conditions would affect the relationship between the SW and PWR integral. Both factors are known to depend on the preload and afterload (Kass and Beyar 1991; Segers et al. 2002), but because they are measured in the heart and aorta, respectively, they are not necessarily equally affected. A mechanically increased afterload was expected to increase both the PWR and SW, and a mechanically reduced preload was expected to reduce both. These expectations were confirmed. Dobutamine has a weak *β*_2_-adrenergic effect, reducing the afterload; however, with a strong *β*_1_ inotropic effect, it was obviously expected to increase the SW and PWR. We used nitroprusside to test the effect of arterial and venous vasodilation impacting both preload and afterload, which reduced the SW and the PWR integral as expected. Metoprolol reduced inotropy and thus SW and PWR, as expected. A mixed linear analysis was conducted to examine if changing conditions significantly affected the relationship between the SW and the PWR integral, and if the relationship varied between animals. This analysis concluded that changing the animal did not have a significant random effect on the slope, indicating that the relationship between SW and the PWR integral is consistent across different individuals. Using the baseline as a reference condition, all the conditions except increased afterload had *P*-value sufficient to assume a fixed effect on the slope. The lowest slope values were produced by metoprolol infusion. The data in this experiment were insufficient to draw any conclusion about why the SW and the PWR integral were affected differently in these conditions; this issue should be addressed in follow-up studies.

Both the SW and PWR integral are measures of the total hydraulic power, which should not be confused with the mean power, which does not include oscillatory power (Westerhof et al. 2005). As both measure the same entity, although at two different locations, the SW and the PWR integral should theoretically have the same value (Westerhof et al. 2005). However, we found that the PWR integral was rather consistently slightly higher than the SW. Measurement error is a possible explanation to keep in mind, although we took great care to calibrate our instruments according to their manuals, and validated the flow meter against another, newer, and fully calibrated flow meter in the laboratory. The observed difference may be at least partially explained by the fact that SW does not include the energy spent on the filling of the left ventricle, whereas the PWR integral contains all the hydraulic energy transferred to the aorta. Looking at the diagrams, this would be represented by the area below the PV loop in the PV diagram. On the other hand, by conducting our aortic flow measurements on the ascending aorta, we missed the flow to the coronary arteries, which theoretically should reduce the size of the PWR integral relative to the SW. The coronary flow is, however, relatively small in systole, and the PWR integral is mainly calculated from systolic flows (see below). Thus, the PWR integral contains virtually all the energy transferred to the aorta, with the exception of the kinetic energy and friction loss, both of which were presumably negligible in our research objects, which had no obstruction in the left ventricular outflow tract (LVOT). We must emphasize that the PWR integral could not be used as a surrogate for SW in patients with aortic stenosis or mitral insufficiency. We do not consider this a weakness of our method as our intention in measuring the PWR integral is to obtain information regarding the energy actually delivered to the central vasculature. Our study demonstrated that the PWR integral follows SW under multiple loading conditions when there are no obstructions in the LVOT, and that the PWR integral seems to have a slightly higher value.

The PWR integral is much more dependent on systole than on diastole because the diastolic flow is practically zero. Some flow could be detected in diastole, partly because of the Windkessel effect, which is likely to be strong in the healthy young animals in our study. Because the flow in diastole is practically zero, PWR is also close to zero in diastole, and as a result, diastolic pressure is not reflected in the PWR integral. This aspect of the PWR integral might be considered a weakness. However, the energy transfer from the heart to the aorta takes place during systole. Hence, the PWR integral appears to be a more physiologically correct representation of this energy than CPO calculated from the CO and MAP.

It should also be noted that the shape of the PWR curve is practically identical to the shape of the flow curve, as the relative variation of the flow was much higher than the relative variation of blood pressure in our research animals. This observation may make it tempting to conclude that the inclusion of arterial pressure is superfluous, but previous studies (Williams et al. 2001; Cohen-Solal et al. 2002; Fincke et al. 2004; Mendoza et al. 2007) showed that the product of pressure and flow, CPO, correlated better with patient outcome than any of the factors from which it was calculated.

Physiologically, CPO, the product of CO and MAP, represents the mean, not the total, hydraulic energy transferred from the heart to the aorta. Based on the following characteristics, the PWR integral may be more informative regarding the energy transfer from the heart to the vasculature. First, the PWR integral is useful on a stroke-to-stroke basis, allowing instant feedback on interventions, in contrast to CPO, which is based on CO measured as an average over several cardiac cycles. Second, the CO measurements vary with regard to when in the respiratory cycle the measurement is performed (Stevens et al. 1985). In the PWR integral, such errors would be easily corrected by calculating the mean of one respiratory cycle, which is made possible by the live visualization of the PWR. Third, the CO measurement is less reliable in awake patients breathing spontaneously, as demonstrated by Kirkeby-Garstad et al. (2008). The authors concluded that this reduction in reliability was due to irregular breathing patterns in awake patients making synchronization of the indicator injection more difficult, and due to the clinical situation as a whole. Finally, in most studies published on CPO, the CO was measured by right heart thermodilution with a pulmonary artery catheter or with gas rebreathing methods. These methods are not available in all clinical departments and may have limited precision in some clinical settings (Kirkeby-Garstad et al. 2008). They are also more invasive than the methods we potentially can use for PWR, for example, ultrasound combined with an arterial catheter.

Similar to the PWR integral, the SW includes the entirety of the mechanical energy from the heart during one cardiac cycle. The SW can, however, not be measured in a minimally invasive manner by the available methods. Also, with obstructions in LVOT, the SW will not yield reliable information regarding the actual energy delivery to the central aorta, due to energy loss over the stenosis caused by turbulence. We have demonstrated that the PWR integral follows the SW where there are no obstructions in the LVOT. The PWR integral can potentially be measured by minimally invasive methods (Sharir et al. 1994; Nakayama et al. 1998; Schmidt et al. 1999), which could open many new possibilities involving the instant monitoring of cardiac energy delivery, such as monitoring the changes in unstable patients and stroke-to-stroke monitoring of the effects of therapeutic interventions against circulatory failure.

The PWR integral might provide a less invasive method to predict when the limit of preload recruitable SW has been reached (Glower et al. 1985), allowing the patient to avoid pulmonary edema and other serious complications from excessive fluid resuscitation. Combined with the monitoring of systemic vascular resistance, we believe that the PWR could also yield an accurate hemodynamic diagnosis in patients with acute congestive heart failure; this expectation is based on the results obtained with the CPO (Cotter et al. 2003).

### Limitations of our study

A possible weakness with our study is that the distance between the flow probe on the ascending aorta and the micromanometer on the proximal descending aorta can introduce an error due to a propagation delay. This distance is, however, very short in our research project because pigs generally have a very short ascending aorta, which is even shorter in piglets. In further studies with a more distal placement of the pressure manometer, this delay can be corrected in the software.

In this study, we only investigated the SW-to-PWR integral relation in healthy hearts. This relation could be different in disease states, which we consider possible subjects for further research.

Regarding clinical use, the PWR integral will require some training to acquire, also if ultrasound Doppler combined with a pressure catheter is demonstrated to be reliable. The ultrasound skill needed would be to measure aortic flow, for instance, through a transthoracic apical window. As ultrasound is becoming more common in emergency rooms and intensive care units, we do not expect the training required to measure the PWR integral to be too demanding.

## Conclusion

In this study, we have validated a system for acquiring the PWR integral as a measure of the energy transferred to the central aorta. The PWR integral followed SW across multiple different loading conditions and across multiple different subjects, the PWR integral was however generally slightly higher than SW. We believe the next natural step is testing a less invasive method for measuring the PWR integral, such as transesophageal Doppler measurements combined with a radial artery catheter. If successful, this method can provide an easily accessible and less invasive method for assessing the energy delivery from the heart to the circulation, allowing it to become a valuable tool in optimizing circulatory status in hemodynamically unstable patients.
